# Relationship Between TNF-α and the Risk of Cerebral Palsy: A Systematic Review and Meta-Analysis

**DOI:** 10.3389/fneur.2022.929280

**Published:** 2022-06-13

**Authors:** Baotian Wang, Fan Wang, De Wu, Xiaoyan Xu, Li Yang, Jing Zhu, Jinjing Yuan, Jiulai Tang

**Affiliations:** Department of Pediatrics, First Affiliated Hospital of Anhui Medical University, Hefei, China

**Keywords:** cerebral palsy, TNF-α - tumor necrosis factor alpha, pathogenesis, meta-analysis, systematic review

## Abstract

**Objective:**

We performed a meta-analysis to investigate the relationship between blood tumor necrosis factor-alpha (TNF-α) levels and the risk of cerebral palsy (CP) in children.

**Methods:**

PubMed, Web of Science, Cochrane Library and Ovid databases were searched from the date of database inception to 26 April 2022. Data were extracted and pooled from observational studies related to TNF-α and the risk of CP in children. Quality was assessed using the Newcastle-Ottawa Scale. We used the inverse variance method with a random-effects model to estimate the odds ratios with 95% confidence intervals (CIs), and stratified analyses and sensitivity analysis were utilized to analyse heterogeneity.

**Results:**

Nine studies with 1,117 cases and 3,563 controls were included in our meta-analysis. The quality of the literature was good, and no publication bias was noted. According to the random-effects model, blood TNF-α levels were associated with the risk of CP (OR 1.82; 95% CI, 1.25–2.66) in a heterogeneous set of studies (*I*^2^ = 81.2%, *p* = 0.000).

**Conclusion:**

Our findings indicate that elevated TNF-α levels in the blood are associated with an increased risk of CP. The association of TNF-α with CP requires further investigation.

## Introduction

Cerebral palsy (CP) is a group of permanent movement and postural disorders that are attributed to non-progressive disturbances in the developing fetal or infant brain (1). CP is the most common form of chronic motor disability in childhood, and the prevalence is 2.11 cases per 1,000 live births (2). A systematic review estimated that among children with CP, “1 in 2 had an intellectual disability; 1 in 4 had epilepsy; 1 in 4 could not talk; 1 in 4 had a behavior disorder; 1 in 10 were blind and 1 in 25 were deaf” (2). Moreover, despite advancements in medical technology in recent years, the incidence of CP has not significantly declined (3). Currently, inflammation, genetic factors and prematurity have been identified as the strongest risk factors for CP; however, in most cases, the etiology of CP remains poorly understood (4). Additionally, because the specific details of the events that lead to CP are often unknown, we cannot derive a causal relation to later neurological dysfunction in humans (4).

Tumor necrosis factor-alpha (TNF-α) is a cytokine that is mainly produced by astrocytes, microglia, blood-borne macrophages, and vascular endothelial cells in the central nervous system and plays important roles in immune modulation, the inflammatory response, cell proliferation and apoptosis. TNF-α directly induces the aggregation of multinuclear leukocytes in an injured area and activate the release of inflammatory mediators, which can be further enhanced by inducing the synthesis of inducible adhesion factors, interleukin (IL)-6, IL-2, and IL-8. Activated microglia can induce blood-brain barrier dysfunction by releasing TNF-α, which participates in the development of cerebral oedema (5). Evidence has shown that repeated increases of the concentration of TNF-α in blood contribute to CP (5–12), whereas other studies, including a meta-analysis (13), reported that there was no statistically significant correlation (13–16). It is hard to define whether the elevation of TNF-α is responsible for the CP or if both processes occur in parallel. Considering these conflicting results, we performed a meta-analysis of published observational studies to investigate whether TNF-α is associated with CP in children. Evidence of a relationship between TNF-α and CP could help illuminate the pathogenesis of this disorder.

## Methods

### Literature Search and Selection Criteria

This meta-analysis adhered to the preferred reporting items for systematic reviews and meta-analyses (PRISMA) guidelines. PubMed, Web of Science, Cochrane Library and Ovid databases were searched systematically from the date of database inception to 26 April 2022. The following keywords were used in our search strategy: (“cerebral palsy”) and (“tumor necrosis factor-alpha” or “TNF-α” or “TNF-a” or “TNF-alpha” or “TNF antagonists” or “TNF inhibitors” or “TNF blockers” or “tumor necrosis factor antagonists” or “tumor necrosis factor inhibitors” or “tumor necrosis factor blockers”). In addition, manual retrieval of references of relevant original articles was performed by two reviewers (BW and FW) to avoid missing additional pertinent studies. We restricted the searches to human studies, and only articles published in English were considered.

Two authors (BW and FW) independently conducted the original search, removed duplicate articles, examined the titles and abstracts of all initially identified studies and performed a full-text evaluation of the selected articles. Any discrepancies between the two authors were resolved through discussion with the senior fellow (DW). The studies were included in the analysis if they satisfied the following criteria: (1) the study described the relationship between TNF-α and CP risk in children; (2) the sample of TNF-α were obtained from blood; (3) the article was published in English; (4) the study was an observational study; and (5) the study provided sufficient data, including odds ratios (ORs) with 95% confidence intervals (CIs). Reviews, meta-analyses, case reports, letters, editorials, animal studies, conference abstracts, simple commentaries and studies without accessible data after contacting the corresponding author were excluded.

### Data Extraction and Quality Evaluation

The following data were independently extracted from each report by two reviewers (JZ and XX): the first author, year of publication, sample size, number of CP cases, definition of and diagnostic criteria for CP, participants' age range, country of origin of the studied population, study duration, study design, gestational age, method of obtaining blood samples, OR for the association between CP and TNF-α and the associated 95% CI and confounders used for model adjustment.

The Newcastle-Ottawa Scale (NOS) (17) was used by two authors (LY and JY) to assess the methodological quality of each study included in the current meta-analysis. The NOS assigns a maximum of nine points to each study. We considered NOS scores of <5 stars, five to seven stars, and more than seven stars as indicators of low, medium, and high quality, respectively. Any discrepancies between the two authors were resolved through discussion with the senior fellow (JT).

### Statistical Analysis

The original studies included ORs and 95% CIs estimating the relationship between TNF-α and the risk of CP in children. We used the random-effects model to calculate both the Q and *I*^2^ statistics as indicators of heterogeneity. We performed subgroup analyses to identify the sources of heterogeneity among the studies. Between-subgroup heterogeneity was assessed using the fixed-effects model. The heterogeneity was considered to be high if the *I*^2^ value was >50% and the *p*-value was <0.1. The value of the *I*^2^ statistic was used to select the appropriate pooling method: fixed-effects models were used for *I*^2^ <50% and random-effects models were applied for *I*^2^ > 50%. Sensitivity analyses were conducted by excluding each study one at a time to examine whether the overall effect estimate changed. Publication bias was assessed using Egger's (18) and Begg's tests (19). A trim-and-fill method was used to further assess the possible effect of publication bias on our meta-analysis (20). We used Stata software v15.1 (StataCorp, College Station, TX, USA) to perform the statistical tests, and *p*-values < 0.05 were considered statistically significant.

## Results

### Literature Search

**Figure 1:**
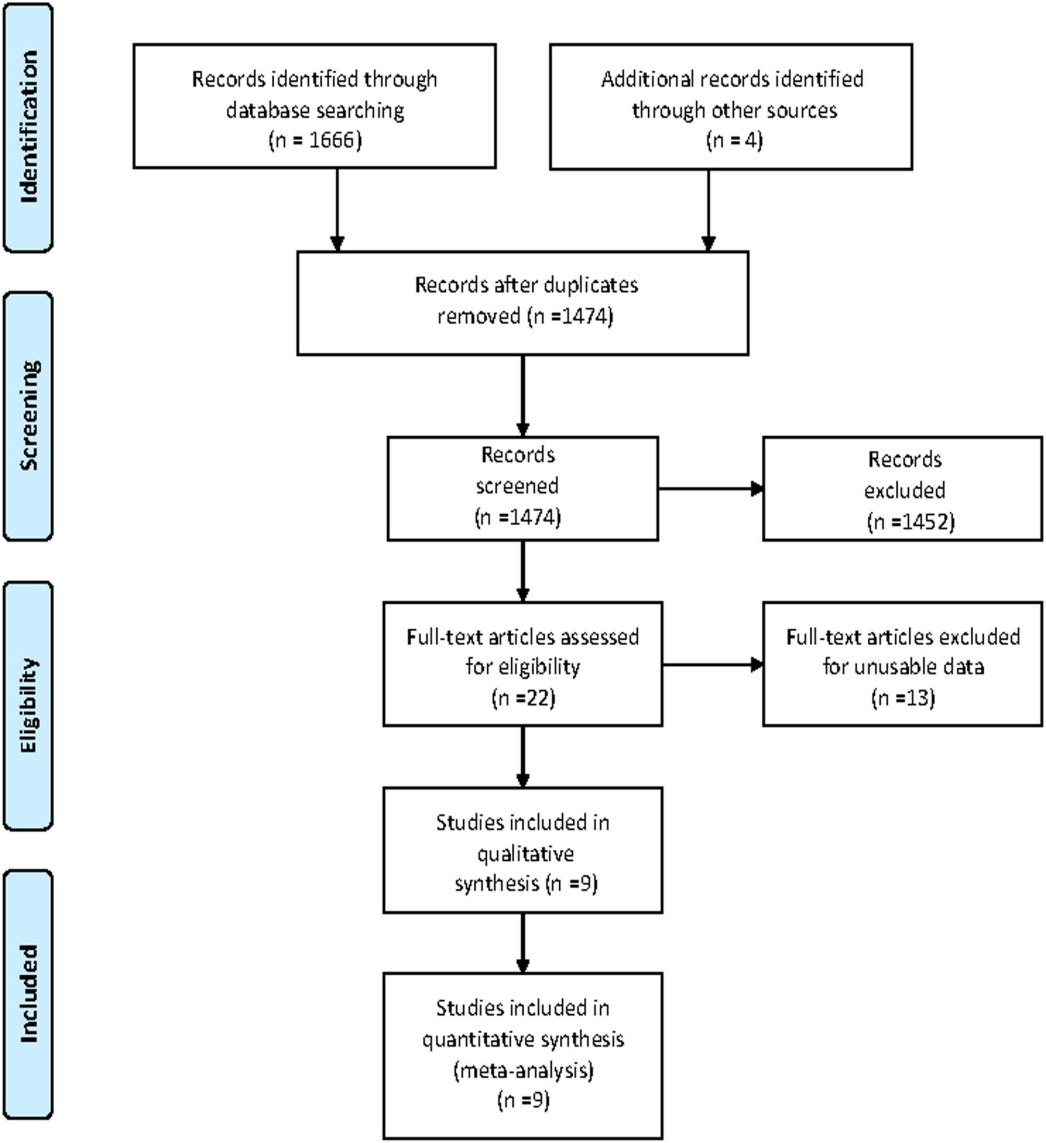
Flow chart for study searching and selection process.

A total of 1,670 articles were retrieved from the databases: 109 articles from PubMed, 1,307 articles from Ovid, 20 articles from the Cochrane Library, 230 articles from Web of Science and 4 additional studies from the related references. After screening the titles and abstracts and excluding duplicates, 22 remaining records seemed to be relevant to this study. After reviewing the full-text articles, nine articles (5–11, 14, 15) were included in this meta-analysis. The flowchart for the search and selection results is shown in Figure 1.

### Characteristics and Quality of the Included Studies

A summary of the study designs and main characteristics of the included studies are shown in Table 1. In total 1,117 cases and 3,563 controls were eligible and included in this study; the sample size ranged from 74 participants (6) to 1,741 participants (15). All included studies were observational in nature, including three cohort studies (6, 7, 10), four case-control studies (5, 8, 9, 14) and one randomized controlled trial (11, 13). Four studies were conducted in the United States (8, 10, 11, 14), two studies were conducted in Europe (6, 7), one study was conducted in Australia, (15) and the others were conducted in China (5, 9). Of the five studies conducted at the beginning of the neonatal period before the diagnosis of CP, two collected blood samples from cord blood (6, 14) and three from venous blood (8, 10, 11), and all four infant studies collected venous blood samples (5, 7, 9, 15). The methods used to assess TNF-α differed among the studies, two studies used Enzyme-linked Immunoassay (ELISA) (5, 14), three studies used SNP analysis (7, 9, 15) and four used cytometry techniques, including Cytometric Bead Array (CBA) (6), Meso Scale Discovery (MSD) electrochemiluminescence system (8, 10), Multiplex Luminex Assay (11). The total NOS scores of the nine included studies ranged from 6 to 9. The ORs with 95% CIs and the NOS quality assessment score for the eight included studies are shown in Table 1.

**Table 1:** Characteristics of the included studies.

**Author**	**Publication year**	**Country**	**Study design**	**Definition and diagnosis**	**Study period**	**Total participants**	**CP cases**	**Patients age**	**Gestational age**	**Test**	**Methods**	**Primary outcome**	**NOS score**
Wu	2015	China	Case-control	Yes	Dec 2012–Aug 2013	82	54	1 y−12 y	NA	ELISA	Venous blood	OR = 1.605 (CI 1.302–1.979)	6
Hansen	2008	Sweden	Cohort study	No	Feb 2001–Feb 2003	74	5	24-months corrected age	GA <32w	CBA	Cord blood	OR = 3.6 (CI 1.002–12.8)	9
Vidak	2012	Croatia	Retrospective study	Yes	NA	144	60	5 y	GA <32w	SNP	Venous blood	OR = 3.286 (CI 1.067–10.116)	8
Yanni	2017	USA	Cohort study	No	2002–2004	763	87	24-months corrected age	GA <28w	MSD	Venous blood 1,7 and 14d	OR = 3.1 (CI 1.2–7.9)	9
Hou	2016	China	Case-control	Yes	2012–2014	219	105	mean age = 24 months	GA≥37w GA <37w	SNP,ELISA	Venous blood	OR = 2.75 (CI 1.23–6.13)	6
Varner	2015	USA	Randomized controlled trail	Yes	Dec 1997-May 2004	609	102	2 y	24 ≤ GA <32	ELISA	Cord blood	OR = 1.05 (CI 0.50–2.21)	9
Kuban	2014	USA	Case-control	Yes	2002–2004	939	105	2 y	GA <28 w	MSD	Venous blood	OR = 3.5 (CI 1.4-8.6)	6
O'Callaghan	2013	Australia	Case-control	Yes	Jul 2008–Mar 2010	1,741	587	5 y−18 y	Average gestational age = 35.3w	SNP	Venous blood	OR = 0.79 (CI 0.62–1.0)	7
Pappas	2020	USA	Randomized controlled trail	No	Jul 2001–Aug 2010	109	12	0 y−7 y	NA	Luminex Assay	Venous blood 0-1d	OR = 1.66 (CI 1.10–2.51)	6

### Meta-Analysis and Subgroup Analyses

**Figure 2:**
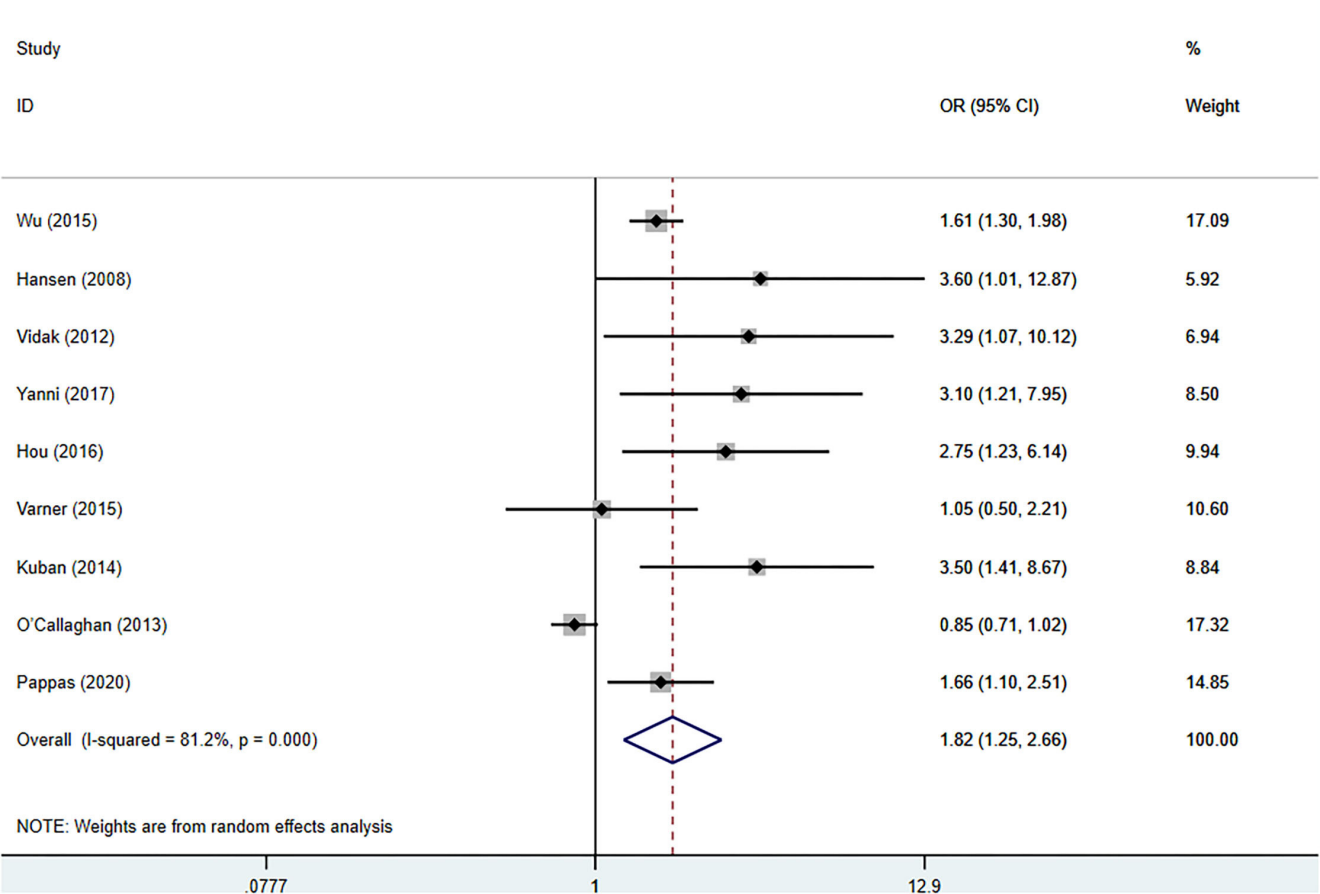
Forest plot of the association between the blood levels of TNF-α and cerebral palsy based on the OR. Weights are obtained from random effects analysis. CI, confidence interval.

The meta-analysis was performed with a random-effects model, and the pooled OR suggested a significant positive relationship between blood TNF-α levels and the risk of CP in children (pooled crude OR 1.82, 95% CI: 1.25–2.66, z = 3.11, *p* = 0.002) with high heterogeneity among the studies (*I*^2^ = 81.2%, *p* = 0.000) (Figure 2). Nine studies evaluated the relationship between TNF-α and the risk of CP in children (5–11, 14, 15), and two reported no significant association (14, 15).

To determine any significant differences in outcomes among the subgroups, we conducted stratified analyses based on a few key study characteristics and clinical factors. The results of all the main and subgroup meta-analyses are shown in Table 2 and Supplementary Figures S1–S5. The relationship between TNF-α and CP risk was not substantially different among any of the subgroups. When stratified by age, the pooled estimate from the included studies revealed a significant positive relationship between blood TNF-α levels and the risk of CP in children (Table 2, Supplementary Figure S2). The definition of CP differed substantially among the included studies, but a significant positive relationship between blood TNF-α risk and the risk of CP in children was still identified (Table 2, Supplementary Figure S5). Six studies (5, 7–9, 14, 15) provided adequate information about the definition of and diagnostic criteria for CP. Some studies confirmed the diagnosis by performing examinations, whereas others did not. Cord blood studies, TNF-α polymorphism studies and studies performed in Western countries did not yield a significant pooled OR. However, a significant relationship was found in venous blood studies, TNF-α studies and studies performed in Eastern countries. This finding indicates that elevated levels of TNF-α in the venous blood are associated with an increased risk of CP in children (Table 2). The age of the included children, the form of TNF-α, the definition of CP, and the ethnicity of the population could affect the relationship between the level of TNF-α in the blood and the risk of CP in children.

**Table 2:** Pooled results of the association between blood TNF-α levels and CP.

**Variables**	**Studies (*n*)**	**OR (95% CI)**	***I*^2^ (*P*-values for heterogeneity)**	*P*-values*
Total	9	1.82 (1.25–2.66)	81.2% (p=0.000)	
**Samples**	
Venous blood	7	1.25 (1.10–1.41)	84.9% (*p* = 0.000)	0.671
Cord blood	2	1.44 (0.76–2.74)	62.7% (*p* = 0.102)	
**Patients age**	
Neonate	5	1.87 (1.38–2.54)	39.2% (*p* = 0.160)	0.005
Infant	4	1.16 (1.02–1.33)	89.4% (*p* = 0.000)	
**Form of TNF-α**	
TNF-α	6	1.68 (1.42–2.00)	30.8% (*p* = 0.205)	0.000
TNF-α polymorphisms	3	0.93 (0.78–1.11)	84.4% (*p* = 0.002)	
**Country**				
East country	2	1.66 (1.36–2.03)	38.2% (*p* = 0.203)	0.001
West country	7	1.07 (0.91–1.24)	79.5% (*p* = 0.000)	
**Definition and diagnosis**				
Yes	6	1.19 (1.04–1.35)	85.3% (*p* = 0.000)	0.012
No	3	1.94 (1.35–2.78)	16.8% (*p* = 0.301)	

^*^Overall test for heterogeneity between subgroups was performed using the inverse variance method.

### Sensitivity Analysis

**Figure 3:**
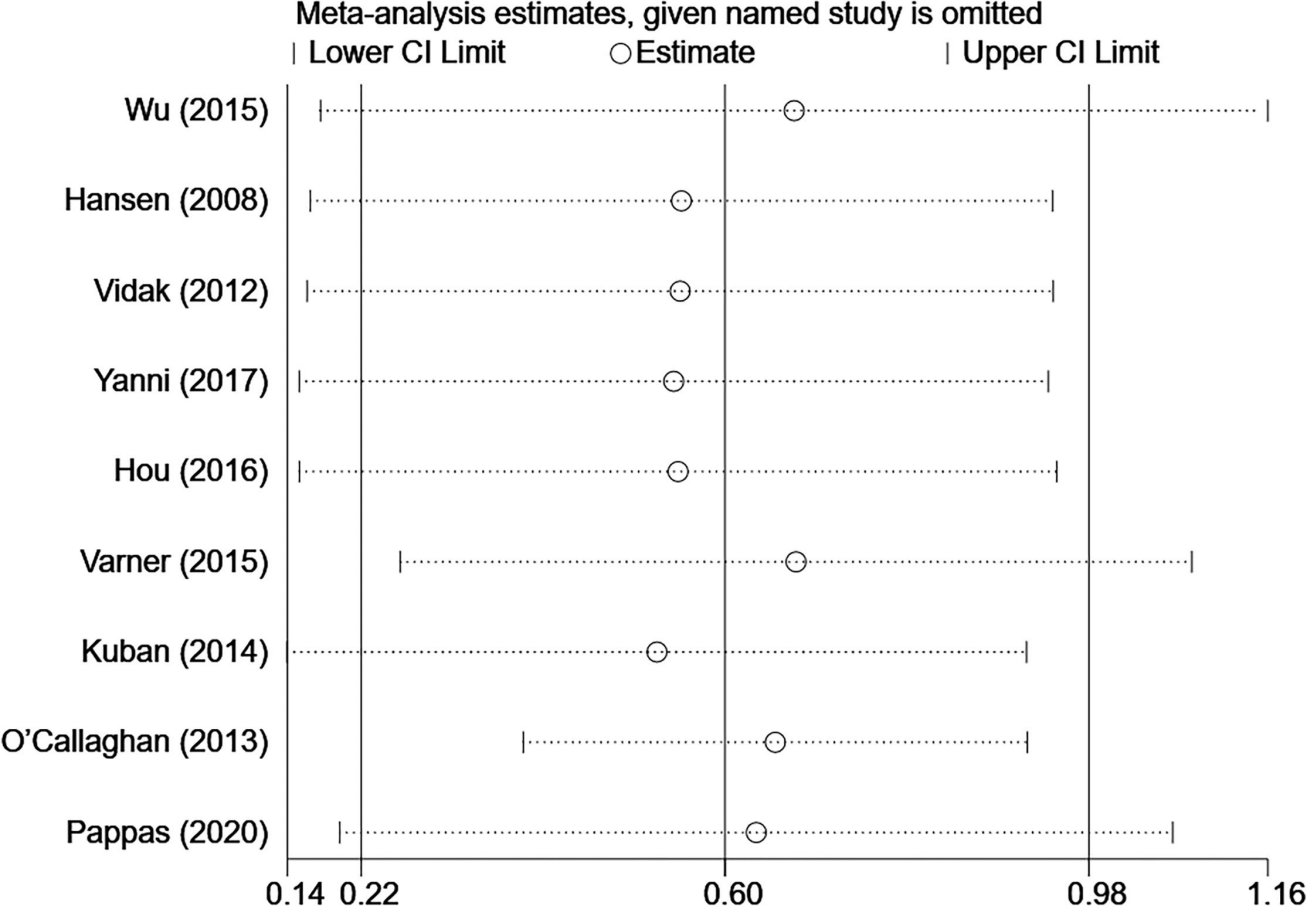
Sensitivity analysis for individual studies on the summary effect.

To confirm the robustness of the meta-analysis, a leave-one-out sensitivity analysis was conducted by recalculating the pooled OR after the exclusion of each study. The heterogeneity among studies changed to 28.8 after eliminating the study by O'Callaghan et al. (15) (OR 1.75; 95% CI, 1.48–2.06) (Supplementary Figure S6). The results show that the corresponding pooled OR was not substantially altered after the exclusion of any single study from the analysis (range of summary estimates: 0.22–0.98), indicating that the results of this meta-analysis are relatively stable and reliable (Figure 3).

### Publication Bias

**Figure 4:**
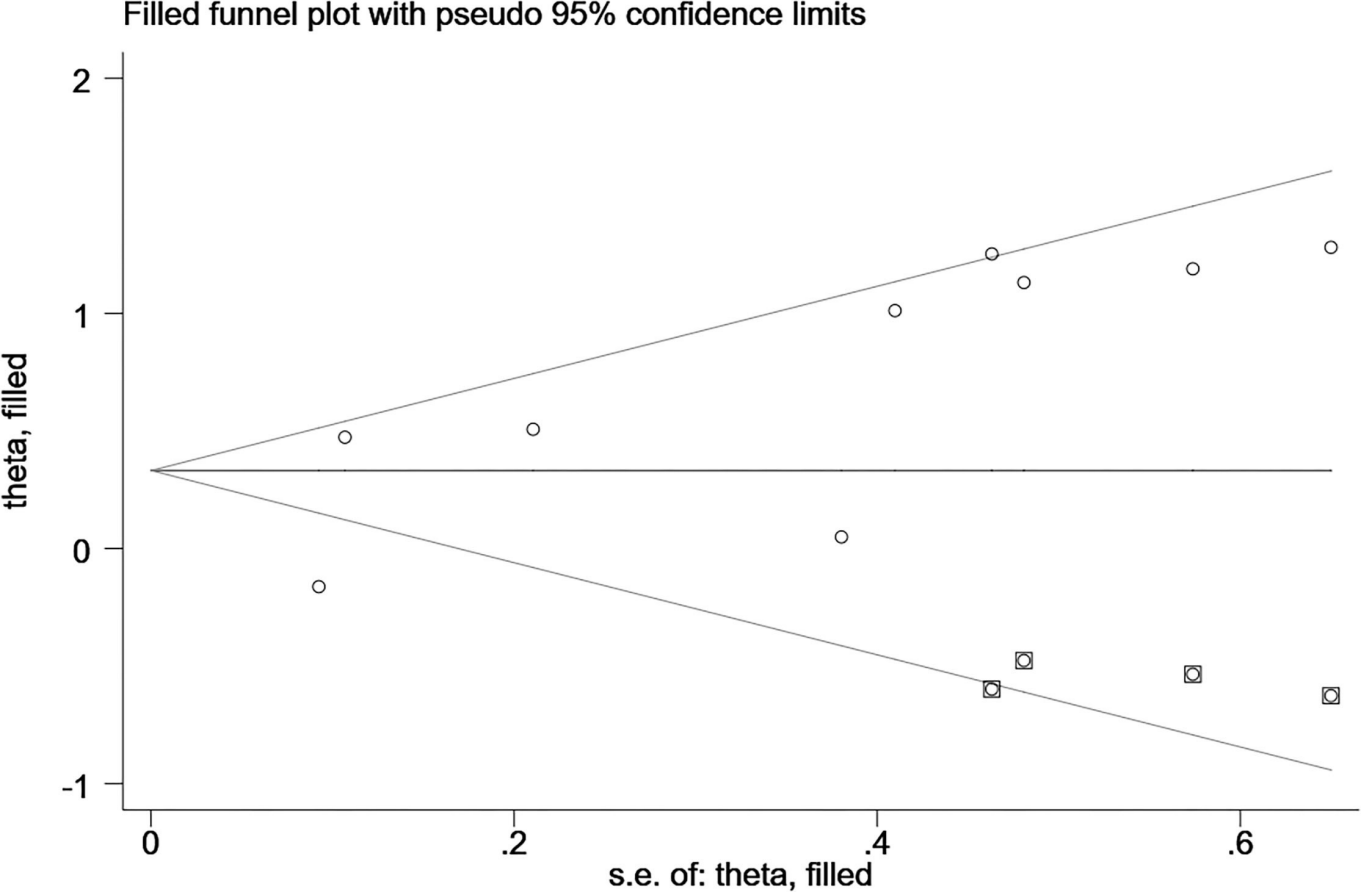
Funnel plots with trim and fill.

**Figure 5:**
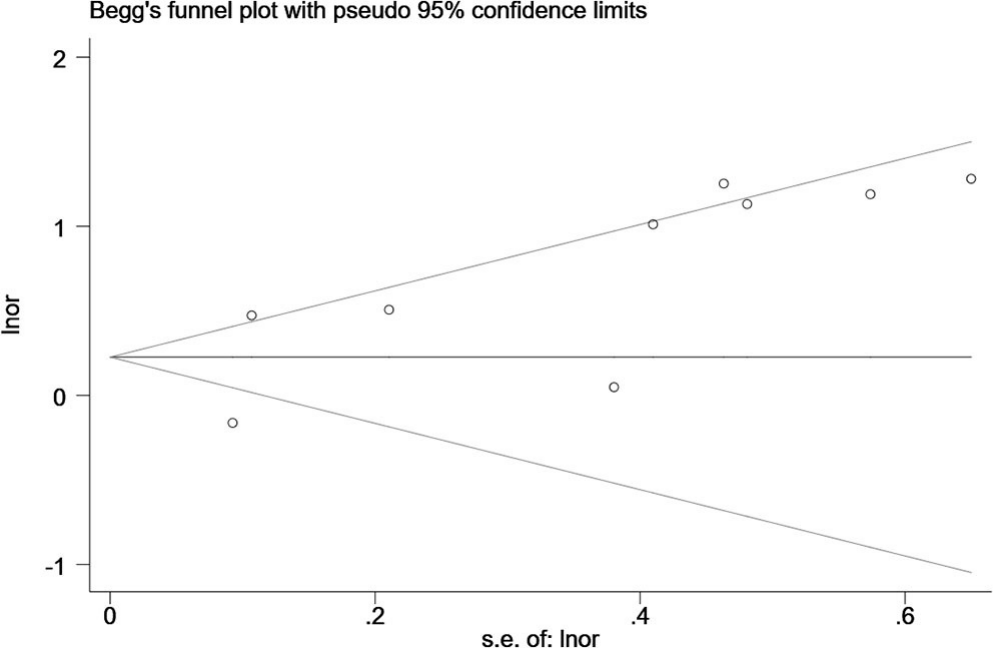
Funnel plots without trim and fill.

Egger's and Begg's tests were used to evaluate funnel plot asymmetry and publication bias. Although no asymmetry was observed in Begg's funnel plot (*P* = 0.92), findings from Egger's test (*P* = 0.04) rejected our null hypothesis about publication bias. Therefore, we performed the trim-and-fill method to recalculate our pooled risk estimate, and the missing results from four studies were imputed, producing a symmetrical funnel plot (Figures 4, 5). The pooled results did not support obvious publication bias (*P* = 0.208).

## Discussion

Our meta-analysis clearly suggests that TNF-α levels in the venous blood are significantly positively related to the risk of CP in children, and the results remained stable in the sensitivity analyses.

**Figure 6:**
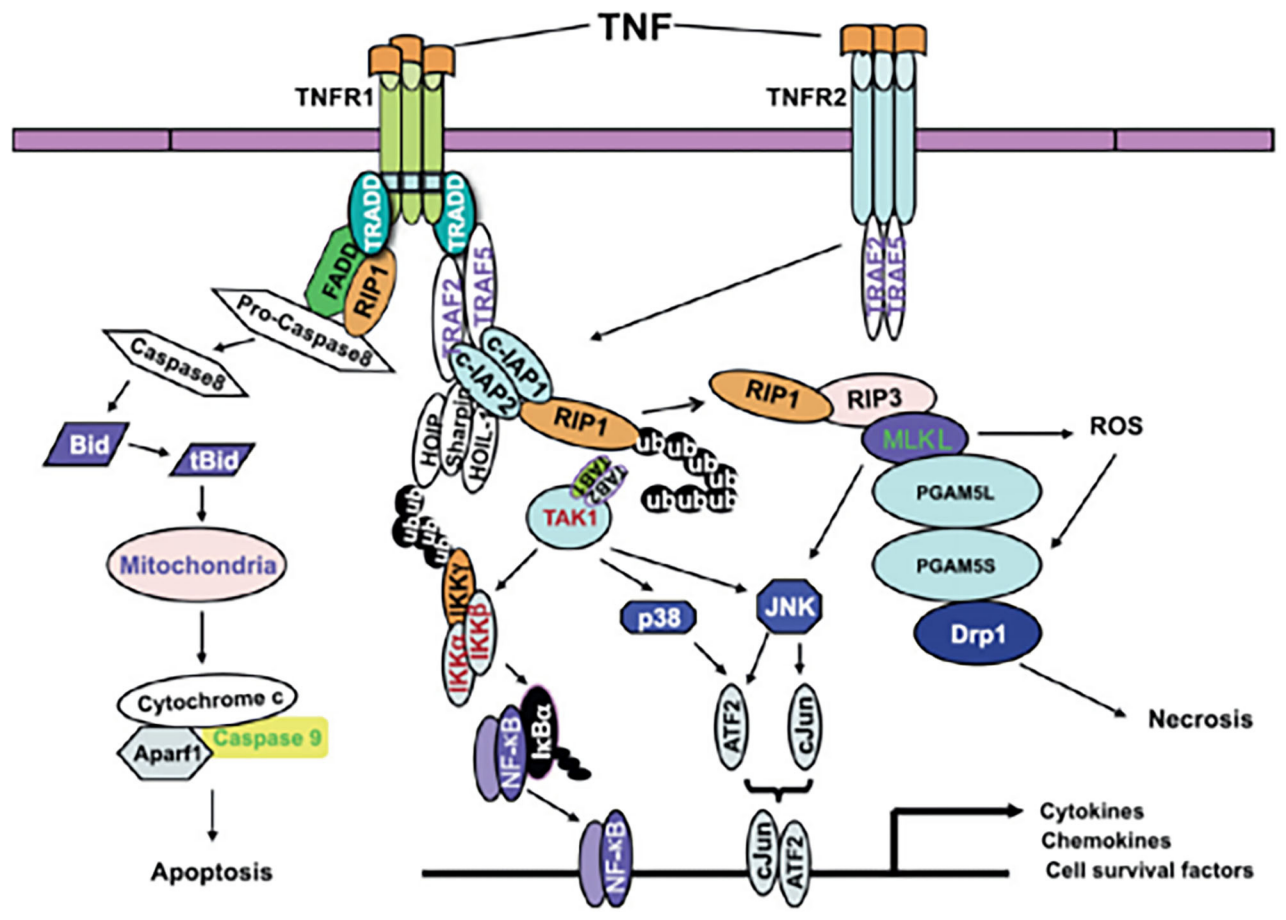
A model of TNF-α signaling in inflammation, apoptosis and necrosis (23).

According to the existing definition, CP describes a group of movement and postural disorders that limit activity and are attributable to non-progressive disturbances in the developing fetal or neonatal brain. It is difficult to explain the relationship between TNF-α and CP. Does CP cause an increase in blood TNF-α levels? Does an increase in blood TNF-α levels cause CP? Alternatively, does an interaction occur between the two factors? Four studies (6, 8, 10, 11) found elevated levels of blood TNF-α in neonates, three studies (5, 7, 9) found elevated levels in infants, and no significant difference was noted between the two groups. According to the existing definition of CP, an increase in TNF-α in the neonatal period is a risk factor for CP. However, it is difficult to explain the increase in TNF-α in the blood of infants with CP. It is important to emphasize that brain development after an inflammation related injury is not a static event, but a complex and dynamic process, in which several cellular and molecular cascades are involved, throughout lifetime (12). To determine the association between TNF-α and CP, it is crucial to know the mechanism underlying brain injury induced by TNF-α. A relationship between TNF-α and CP in children has long been hypothesized. Because these molecules can cross the blood-brain barrier (BBB), the peripheral levels of these molecules may also reflect their levels in the brain (12). Based on the involvement of tertiary damage mechanisms (4) and a multi hit model (21, 22) brain injury processes can persist for many months or years. Multiple hits and tertiary damage mechanisms implicated in the pathogenesis of this condition include microglial activation and disturbances of apoptosis, resulting in the arrest of oligodendrocyte maturation and impaired neurogenesis, axonal growth, and synaptogenesis (21). Repeated or long-term chronic stimulation can lead to microglial aggregation and continuous activation and the release of many pro-inflammatory factors, which can generate a “waterfall” effect by activating other microglial cells and amplifying the inflammatory cascade reaction. Thus, a vicious cycle resulting in brain injury occurs. TNF-α is one of the most important pro-inflammatory cytokines, and it plays pivotal roles in systemic inflammation, apoptosis and necrosis (Figure 6 (23)). TNF-α inhibits the differentiation of oligodendrocyte progenitor cells, lead to white matter injury (24), modulate synaptic connectivity and facilitates glutamate-dependent neuron death, which may have a damaging effect on gray matter structures (5) and contribute to brain injury. CP may be caused by undetectable progressive brain injury, and TNF-α may be involved.

Another early meta-analysis did not detect an association between the TNF-α gene rs1800629 polymorphisms and CP (13), which was different from our finding that elevated blood TNF-α levels are related to an increased risk of CP. The relationship between TNF-α gene polymorphisms and the risk of CP in children remains controversial. Vidak et al. (7) and Hou et al. (9) believed that TNF-α gene polymorphisms were significantly associated with CP; however, Wu et al. (13) and O'Callaghan et al. (15) reported the opposite result. Our meta-analysis suggested that the age of the included children, the form of TNF-α, the definition of CP, and the ethnicity of the population could affect the relationship between blood TNF-α levels and the risk of CP in children. Although our results indicates high heterogeneity among the studies, when we omitted the study by O'Callaghan et al. (15) from the analysis, the heterogeneity index decreased to a low level (Supplementary Figure S6). The changes did not alter the statistical or clinical significance of the original findings. Therefore, we suggest that the results are robust. Because the development of CP involves inflammation, these findings may affect the long-term health of children. It is important to emphasize that brain development after an inflammation related injury is not a static event, but a complex and dynamic process, in which several cellular and molecular cascades are involved, throughout lifetime (12).

However, some studies have reported no relationship between the level of TNF-α in cord blood and the risk of CP in children (14). What is the mechanism by which TNF-α levels are not be elevated in the cord blood immediately after birth but become elevated in the venous blood after birth? The mechanism underlying the elevated plasma levels of TNF-α in children with CP may partly be related to an increase in the secretion of TNF-α by peripheral blood mono-nuclear cells (24). Therefore, it is possible that the level of TNF-α in fetal blood could be elevated, but this elevated level is not detectable in cord blood at the time of sampling (25).

A recent paper by Pham et al. (26) expressed concern that most published genetic studies of CP poorly or inadequately defined their inclusion criteria for CP. According to the Pham et al. study (26), limited compliance with international consensus guidelines regarding the phenotypic definition of CP and mediocre reporting of CP case ascertainment criteria hinders the comparison of results among genetic studies of CP (including meta-analyses), thereby limiting the quality, interpretability, and generalizability of study findings (26). Our results also suggest that the definition of and diagnostic criteria for CP could affect the relationship between blood TNF-α levels and the risk of CP in children. A subgroup analysis of studies stratified based on description of these aspects found that there were differences in the results (*P* = 0.01); however, the main finding that blood TNF-α levels were associated with the risk of CP remained the same (Table 2, Supplementary Figure S5).

The results of our meta-analysis indicate that the level of TNF-α in children is significantly positively related to the risk of CP, regardless of age (Supplementary Figure S2). Moreover, the present study suggests that the severity of CP is correlated with the TNF-α level in the blood (5). Based on these findings, future studies should focus on elucidating the mechanisms underlying TNF-α-induced brain injury, which may provide additional information about the relationship between blood TNF-α levels and the risk of CP in children.

Figure 6 A model of TNF-α signaling in inflammation, apoptosis and necrosis (23).

To the best of our knowledge, our study is the first meta-analysis to provide evidence that blood TNF-α levels are significantly positively related to the risk of CP in children. However, several limitations should be taken into account. First, the studies used different time-points in their research, impairing the comparison of their findings. Second, we only included studies published in English. Third, the involved studies were all observational in design, it is also difficult to obtain homogenous groups in observational studies and the number of included studies was small. Finally, the bias inherent in observational studies is not eliminated in a quantitative synthesis. In fact, some preterm studies already performed on a subgroup. This already biases our outcome as you know preterm birth is a risk factor for higher inflammation and diagnosis for CP.

## Conclusions

In conclusion, our pooled analyses provide evidence that elevated blood TNF-α levels are related to an increased risk of CP. However, the causal pathway underlying this relationship remains to be elucidated. Hence, future studies that explain the effects of TNF-α on CP are needed.

## Data Availability Statement

The raw data supporting the conclusions of this article will be made available by the authors, without undue reservation.

## Author Contributions

BW: conceptualization, methodology, software, formal analysis, visualization, and writing—original draft preparation. FW, XX, and JZ: data curation. DW: methodology, formal analysis, and supervision. LY: software, formal analysis, and visualization. JY: software and visualization. JT: project administration and writing—reviewing and editing. All authors have read and approved the manuscript.

## Conflict of Interest

The authors declare that the research was conducted in the absence of any commercial or financial relationships that could be construed as a potential conflict of interest.

## Publisher's Note

All claims expressed in this article are solely those of the authors and do not necessarily represent those of their affiliated organizations, or those of the publisher, the editors and the reviewers. Any product that may be evaluated in this article, or claim that may be made by its manufacturer, is not guaranteed or endorsed by the publisher.
