# Supplementary figures and images for: Relationship Between TNF-α and the Risk of Cerebral Palsy: A Systematic Review and Meta-Analysis
Source: Front Neurol. 2022 Jun 13;13:929280. doi: 10.3389/fneur.2022.929280 (PMC9234274; doi:10.3389/fneur.2022.929280)

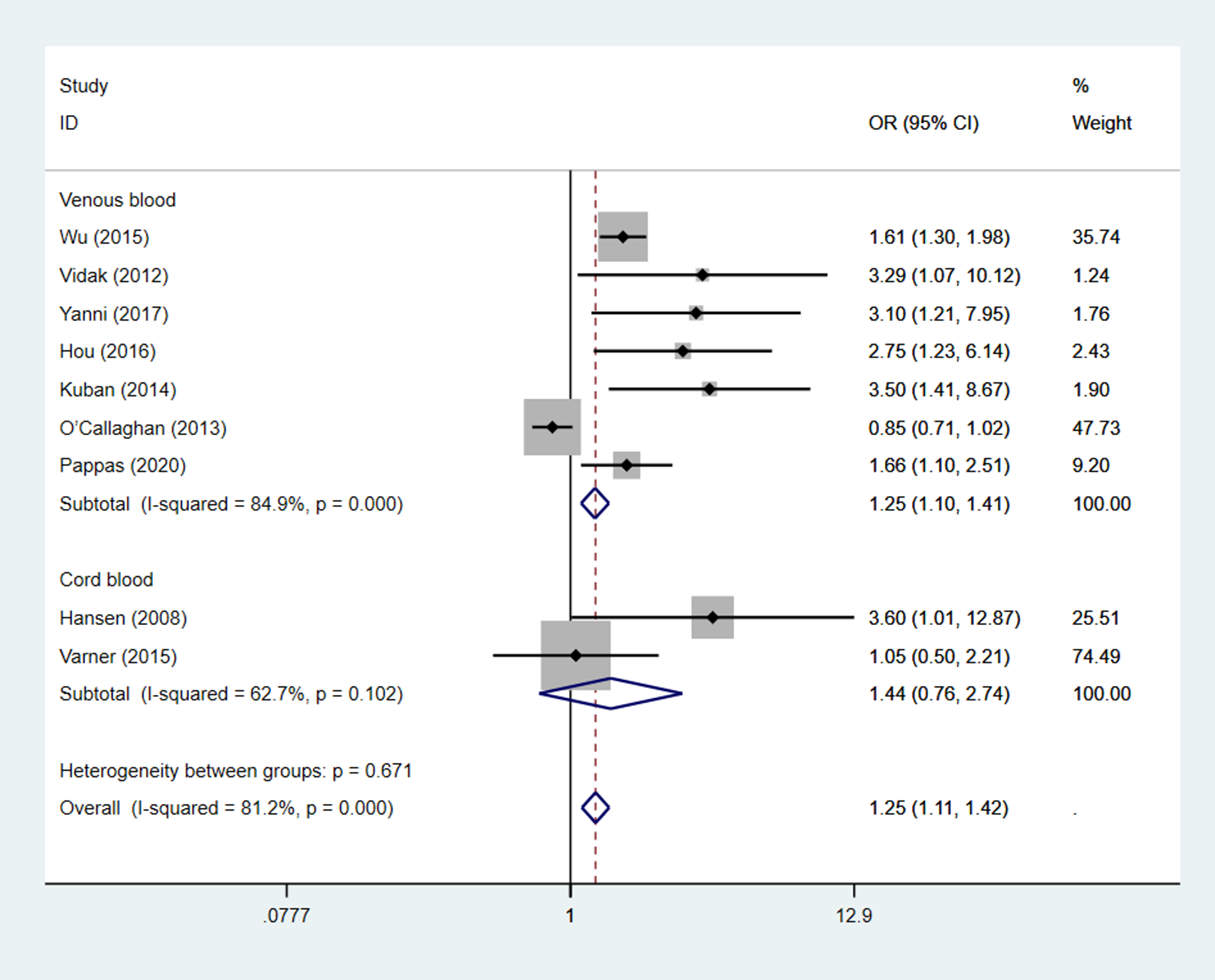

Supplement: Supplementary file 1 [file Image_1.TIF]

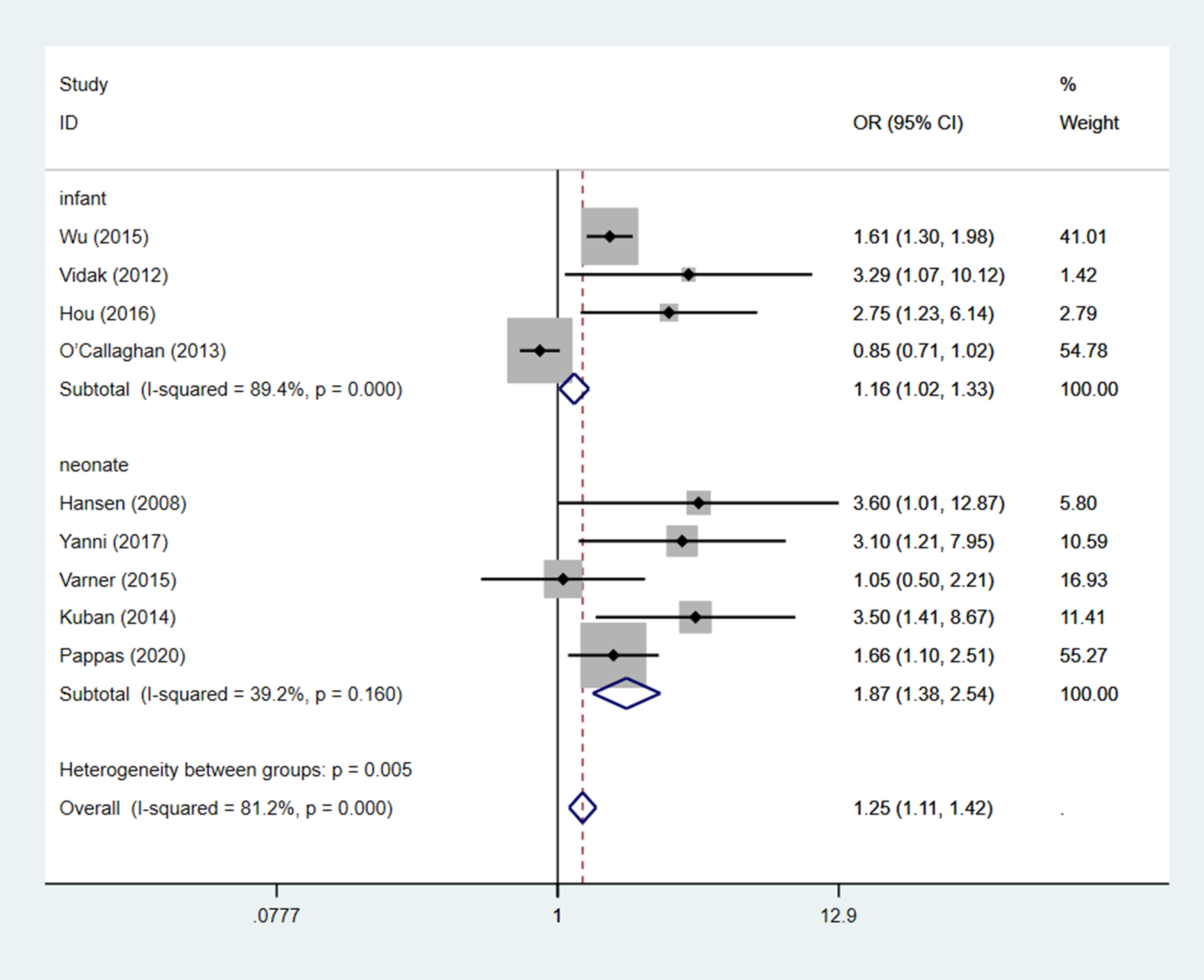

Supplement: Supplementary file 2 [file Image_2.TIF]

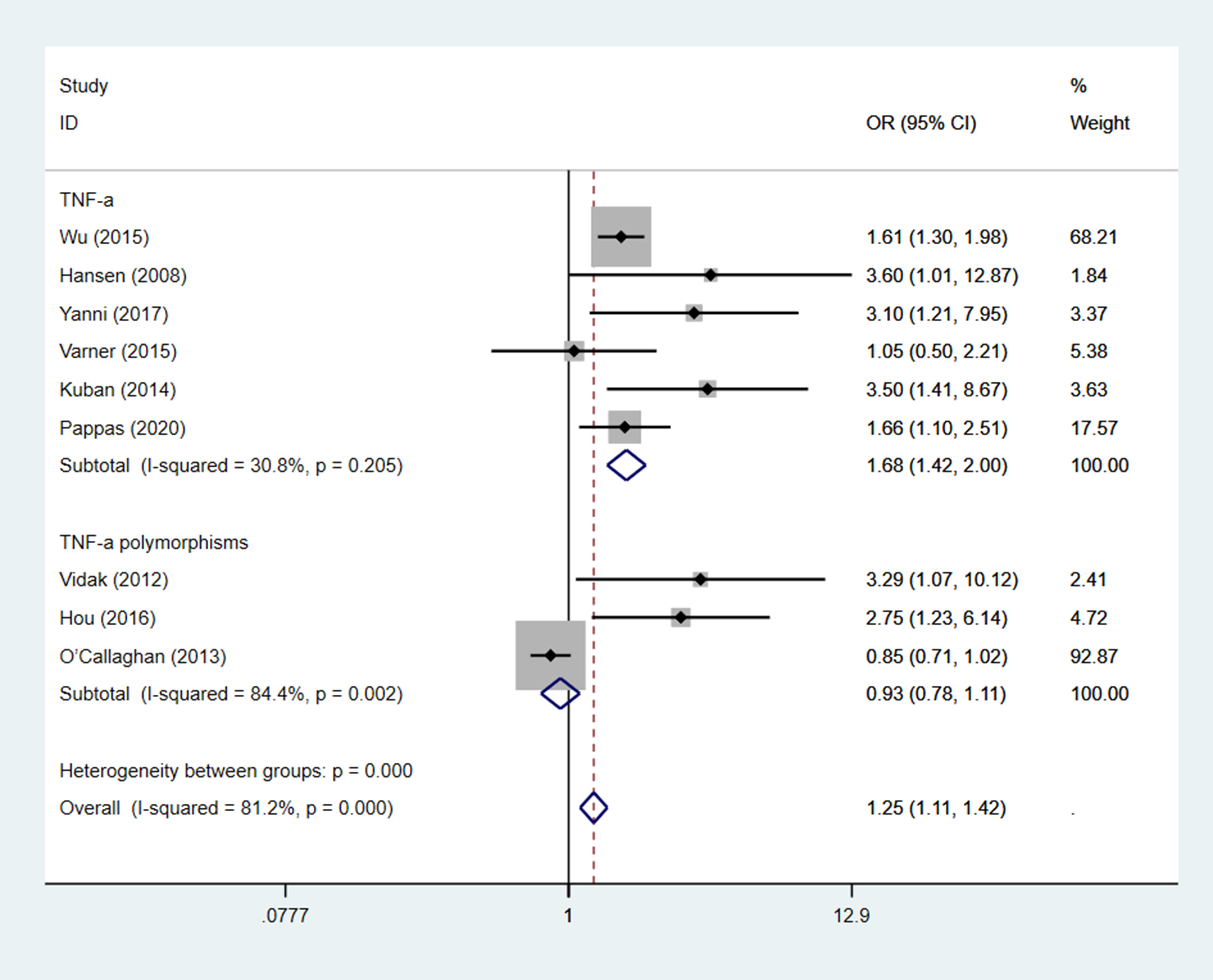

Supplement: Supplementary file 3 [file Image_3.TIF]

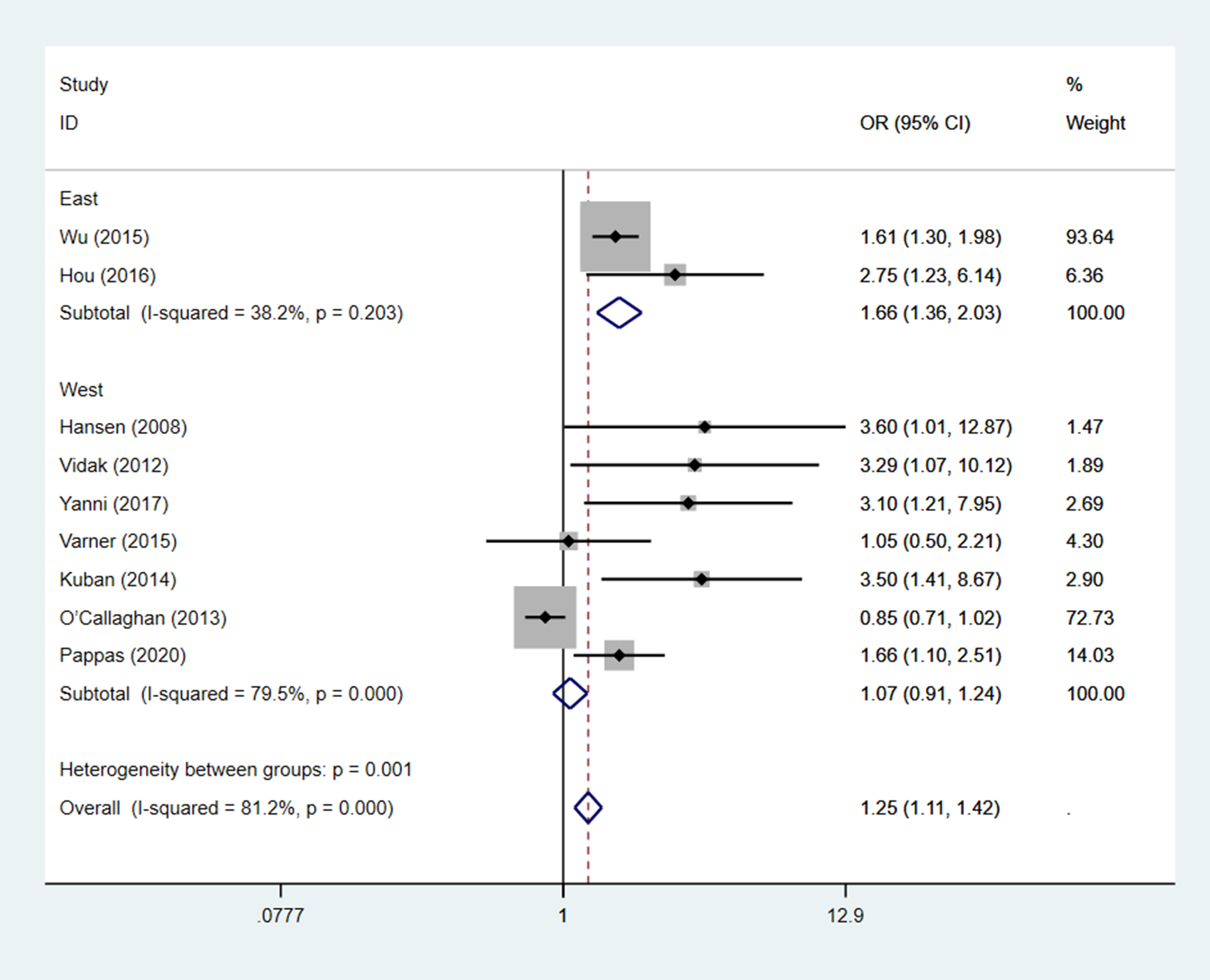

Supplement: Supplementary file 4 [file Image_4.TIF]

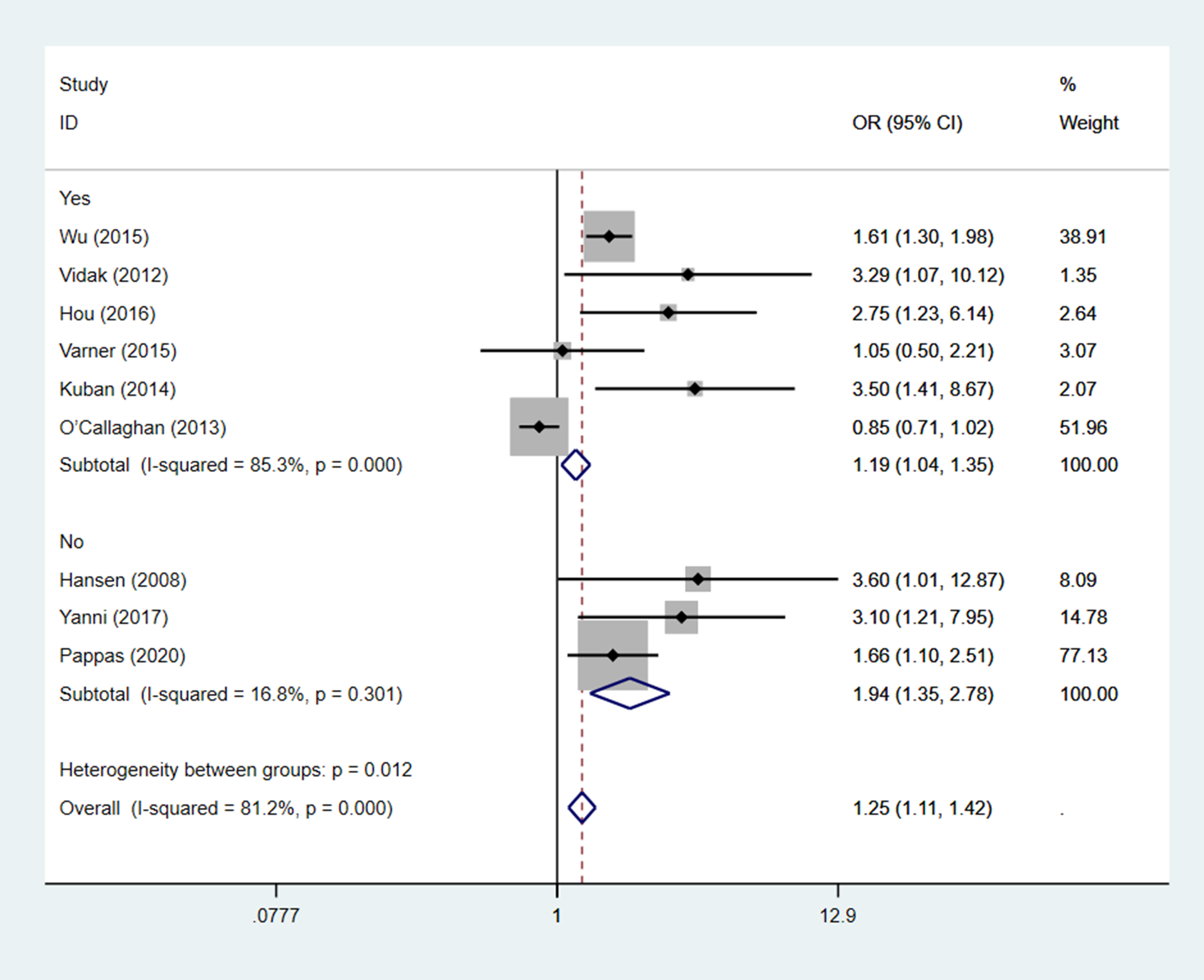

Supplement: Supplementary file 5 [file Image_5.TIF]
